# Corticosteroids: way upstream

**DOI:** 10.1186/1756-6606-3-2

**Published:** 2010-01-11

**Authors:** Therese Riedemann, Alexandre V Patchev, Kwangwook Cho, Osborne FX Almeida

**Affiliations:** 1 Max-Planck-Institute of Psychiatry, Kraepelin Str. 2-10, 80804 Munich, Germany; 2 Henry Wellcome Laboratories for Integrative Neuroscience and Endocrinology, Faculty of Medicine and Dentistry, University of Bristol, Bristol, UK

## Abstract

Studies into the mechanisms of corticosteroid action continue to be a rich bed of research, spanning the fields of neuroscience and endocrinology through to immunology and metabolism. However, the vast literature generated, in particular with respect to corticosteroid actions in the brain, tends to be contentious, with some aspects suffering from loose definitions, poorly-defined models, and appropriate dissection kits. Here, rather than presenting a comprehensive review of the subject, we aim to present a critique of key concepts that have emerged over the years so as to stimulate new thoughts in the field by identifying apparent shortcomings. This article will draw on experience and knowledge derived from studies of the neural actions of other steroid hormones, in particular estrogens, not only because there are many parallels but also because 'learning from differences' can be a fruitful approach. The core purpose of this review is to consider the mechanisms through which corticosteroids might act rapidly to alter neural signaling.

## The protagonists and their roles

Corticosteroids are the main humoral mediators of stress and their increased secretion in response to adverse stimuli normally results in a cascade of physiological and behavioral homeostatic mechanisms that allow survival and the activation of defense mechanisms against future insults. They facilitate arousal and the appropriate channeling of physiological resources; primarily, corticosteroids act to conserve essential salts, stimulate gluconeogenesis and lipid metabolism, cardiovascular and pulmonary function and erythropoeisis and bone turnover, while inhibiting, among others, reproductive and ingestive behaviors as well as immune responses [1]. Thus, corticosteroids are well suited to serve the fight-or-flight response (first described by Walter B. Cannon in 1915).

Corticosteroids (CS) are primarily produced by the adrenal glands although recent studies suggest that they may also be synthesized in the brain [2,3]. The term 'corticosteroids' embraces two prototypic steroids with distinct biological functions: glucocorticoids (cortisol in most large mammals, corticosterone in rodents and other taxa), named because of their gluconeogenic properties, and mineralocorticoids (primarily aldosterone), named for their role in the regulation of the salt-water balance. Like other steroid hormones, corticosteroids are small, lipophilic molecules (ca. 300 Da) that are derived from cholesterol. Their physical properties facilitate their passage across the blood brain barrier where they act to maintain brain structure (they are implicated in the regulation of neuronal cell birth, differentiation and apoptosis, as well as dendritic arborization and synaptic function), and integrate a variety of behavioral and physiological processes, including their own secretion. In this respect, they serve as messengers between the periphery and brain, but also between the external and internal environments and the brain.

**Figure 1:**
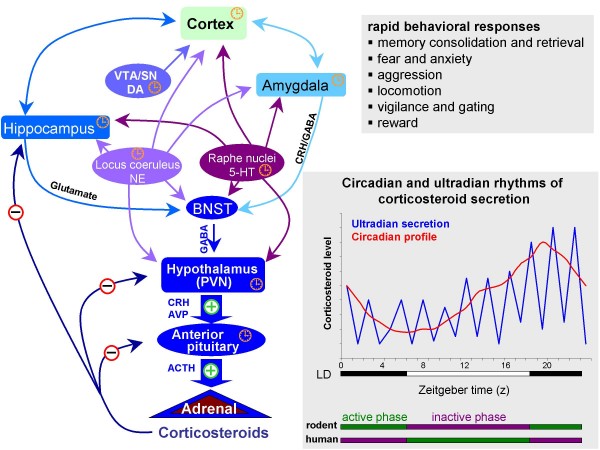
**Schematic representation of the hypothalamo-pituitary-adrenal (HPA) axis and its neuronal inputs**. Corticotropin-releasing hormone (CRH)- and arginine vasopressin (AVP)-expressing parvocellular neurons in the paraventricular nucleus (PVN) project to pituitary (via the median eminence) where they stimulate adrenocorticotrophic hormone (ACTH) synthesis and secretion, subsequently triggering corticosteroid synthesis and release from the adrenal cortex. Besides acting in the brain to regulate various behaviours, corticosteroids fine-tune the subsequent pattern (amplitude and duration) of corticosteroid secretion; they activate their cognate receptors in the pituitary, hypothalamus and hippocampus and bed nucleus of the stria terminalis (BNST, a relay between the hippocampus/amygdala and the PVN) to restrain, and in the amygdala to enhance, adrenocortical secretion. Monoaminergic transmitters, namely, norepinephrine, serotonin and dopamine released from midbrain nuclei (the locus coeruleus [LC], raphé and ventral tegmental area [VTA] and substantia nigra [SN], respectively) exert modulatory effects on all brain regions involved in the control of the HPA axis. 'Plus' signs *(green) *indicate positive drive on the HPA axis; 'minus' signs *(red) *represent sites of corticosteroid negative feedback; 'clock' signs denote neuronal populations known to respond rapidly to corticosteroids. Corticosteroids are secreted rhythmically, displaying ultradian and circadian patterns. The circadian peak coincides with the onset of the daily activity cycle (dark phase in rodents, light phase in humans). While the physiological and behavioural significance of the ultradian rhythms of corticosteroid secretion is still unclear, it is plausible that they serve to dynamically fine-tune the regulation of the HPA axis and thus, to facilitate adaptive processes. *LD*, light-dark cycle.

The hypothalamo-pituitary-adrenal axis embraces the feedforward and feedback neuroendocrine mechanisms that regulate CS production and synthesis (Figure 1). Neural inputs trigger the release of adrenocorticotrophic hormone (ACTH) from the pituitary which, in turn, stimulates adrenocortical synthesis and secretion of CS. Although CS are not stored in a readily-releasable pool, it is estimated that adequate amounts of CS can be released into the bloodstream within minutes of appropriate neural stimuli. Noxious (stressful) stimuli are the primary triggers of neural firing that result in increased CS release. On the other hand, CS are secreted according to strictly-regulated circadian rhythms that are dictated by the central nervous system. More recently, CS have been found to have ultradian rhythmic patterns of release. Such patterns are most likely maintained through dynamic cross-talk between the peripherally-produced CS and centrally-driven regulatory mechanisms; they are also likely important integrators of normo-physiological functions [4].

Since corticosteroids come on stage within 3-7 minutes of first perception of a stressor [5], they may be considered to be secondary or auxiliary players in comparison to monoamines (in particular, epinephrine and norepinephrine) whose actions are initiated within milliseconds to seconds [6] i.e. corticosteroids are secreted during the first stage of the 'general adaptation syndrome', a concept introduced by Hans Selye in 1946. However, since corticosteroids act against the background of increased monoamine secretion, it is thought that they act to fine-tune the organism's response to stress [7] and to facilitate signal-to-noise discrimination. Moreover, unlike the transient monoamine response, corticosteroids exert sustained actions on cellular activity and behavior, and therefore are essential for ensuring the orchestration of a coordinated adaptive response as well as 'preparedness' of the organism to cope with future challenges.

Although corticosteroids are often thought of in negative terms because of their causative role in diseases such as diabetes, hypertension, osteoporosis and immune suppression, they are essential for adaptation to stress and for maintaining physiological processes. With respect to brain structure and function, corticosteroids play an important role in maintaining hippocampal cell numbers under basal conditions; this is illustrated by robust observations that removal of corticosteroids by extirpation of the adrenal glands results in massive apoptosis, with parallel increases in neurogenesis, within the granule cell population of the hippocampus [8]. On the other hand, stress and elevated levels of glucocorticoids inhibit the generation of new granule neurons [9]. Another aspect that suggests an important role of corticosteroids in normo-physiology is the well-pronounced circadian pattern of corticosteroid secretion. These rhythms are robust and bi-directionally tightly coupled to the individual's sleep-activity and feeding cycles, while being entrained and maintained by the daily light-dark cycle.

The magnitude and duration of the humoral response to stress is tightly coupled to the nature (quality, intensity and duration) of the stressor, as well as the context in which it occurs. Depending on context (e.g. the prevailing physiological or psychological state, as well as history of the individual), stressors may trigger excessive corticosteroid secretion over an extended duration; in such cases, the response switches from being an adaptive one into a maladaptive one, marked by transient or chronic pathology. Major depression and cognitive impairment are two conditions that represent the so-called stress-induced disorders of the brain. The first of these seems to reflect a sub-optimal stress-coping strategy and may largely originate from impairments of the mechanisms contributing to the homeostatic negative feedback processes that act to protect the organism against excessive exposure to corticosteroids; frequently, depressed mood is accompanied by impaired cognition and hyperemotionality, indicating that stress impacts on multiple, inter-related neural circuits. A number of human and animal studies have demonstrated the disruptive effects of excessive corticosteroid secretion on cognition [10-12]. There is now strong evidence that the latter involve structural changes, including severe reductions in the dendritic arborization of hippocampal and prefronto-cortical neurons [13-15]. and synaptic loss [16-18]. In addition, recent studies indicate that stress may initiate neurodegenerative processes that increase the risk for severe cognitive deficits such as those seen in dementia of the Alzheimer type [19]. Lastly, chronically elevated levels of corticosteroids interfere with central and pituitary integrators and regulators of the hypothalamo-pituitary-adrenal (HPA) axis, resulting in impaired corticosteroid negative feedback and sustained corticosteroid secretion [20].

## The soliloquy we've come to know and love

Glucocorticoids and mineralocorticoids fulfill their characteristic biological functions through the mediation of glucocorticoid receptors (GR) and mineralocorticoid receptors (MR), respectively. Both of these receptors are present in the brain; while GR are expressed ubiquitously (most strongly in the hippocampus), MR are more discretely distributed (strongly expressed in certain hippocampal subfields and the septum, and moderately expressed in the amygdala and hypothalamic paraventricular nucleus) [21]. The MR has a 7-10-fold greater affinity for corticosterone as compared to the GR [22]. It is thus estimated that the MR is some 80% occupied under basal conditions, and that the GR only becomes activated when corticosterone levels rise during the daily circadian peak of corticosterone secretion or after stress. Although aldosterone may be synthesized in the brain [2,3], it should be noted that brain MR do not normally 'see' their prototypic endogenous ligand; aldosterone is produced in the periphery at concentrations that are too low to have a direct impact on the brain and in any case, the hormone does not easily cross the blood-brain barrier. On the other hand, it should be mentioned that ligand availability is subject to local regulation through activation/deactivation of cortisol/corticosterone through the actions of 11β-hydroxysteroid dehydrogenase [23].

The MR and GR belong to the phylogenetically ancient superfamily of nuclear receptors, all of which are transcriptional factors. For the sake of clarity, we will herein refer to nuclear MR and GR as nMR and nGR, respectively. Whereas the unliganded nMR is primarily localized in the nucleus, the unoccupied nGR resides in the cytoplasm and only translocates to the nucleus upon ligand activation. This process depends on the dissociation of a host of chaperone and co-chaperone molecules, including heat shock protein 90 (hsp90) as well as on the inclusion of a nuclear translocation signal in the receptor protein [24]. Like other nuclear receptors, nMR and nGR are organized according to canonical modules, including a ligand binding domain (LBD), a DNA binding domain (DBD), and two activation functions (AF-1 and AF-2) at their N- and C-terminals, respectively. The various domains share considerable homologies (homology between nMR and nGR: ~57% in LBD; ~94% in DBD). Interactions of the DBD with hormone response elements (HRE) in the promoters of specific genes result in the induction or repression of gene transcription and subsequently, changes in the expression of proteins that influence cellular functions. Homologies also exist within the HRE sequence of various nuclear receptors, and receptor recruitment and interactions with specific co-regulator proteins (co-activators/-repressors) may endow these structurally similar receptors with differing specificities and potencies.

## Stage props

Transcriptional and translational effects of corticosteroid receptor activation have been demonstrated using drugs such as actinomycin D and cycloheximide, respectively. On the other hand, demonstration that nGR mediate corticosteroid effects have relied on the use of the antagonist mifepristone (RU 38486, also a potent antagonist of progesterone receptors), while spironolactone or oxoprenoate (RU28318) have been used to demonstrate mediation through nMR. Other potentially useful additions to the pharmacological toolbox for studying events mediated by nGR and nMR include established chaperone inhibitors of hsp90 (e.g. cisplatin and geldanamycin; [25]) and of the FK506-binding proteins (e.g. GPI1046; [26]).

## Drop scene^b^

The mode of action of corticosteroids summarized above, i.e. involving gene transcription and translation, may be generalized to all steroid hormone receptors, including those for estrogens. Since nuclear receptors become transcriptionally active upon ligand activation, their actions are, by definition, slow in onset and potentially long-lasting (hours to days, or even months); at best, gene transcription and translation require a minimum of 20-30 minutes (translation takes longer than transcription) [27]. However, steroids have been implicated in the elicitation of a number of 'rapid' or 'fast' physiological and behavioral responses to external stimuli; some examples of fast steroid-mediated responses and the mechanisms thought to underlie their actions are presented in Additional File 1. Historically, the idea that steroids can rapidly alter neuronal excitability and conduction stemmed from work on the actions of sex steroids by Kawakami and Sawyer in 1959 [28] and Woolley and Timiras in 1962 [29].

As a rule, fast responses are considered to be those that occur within the first 20 minutes of increased steroid secretion, i.e. in a much shorter timeframe than that required for effects on gene transcription and protein synthesis. Somewhat erroneously, these fast actions are referred to as 'non-genomic'; in fact, rapidly triggered signaling cascades may ultimately converge in the nucleus to regulate gene transcription and protein synthesis. Distinction between the 'fast' and 'slow' actions of steroid hormones is more of mechanistic than of behavioral or physiological importance, since the latter are the integrated manifestations of sequential events. Viewed from this perspective, the rapid actions of steroids may be considered as 'primers' of the substrates responsible for the manifestation of transcriptional events triggered by nuclear receptors; kinase cascades activated during early phases of steroid action and which lead to the phosphorylation of regulatory sites of nuclear receptors [30-32] are a good example of such priming functions.

**Figure 2:**
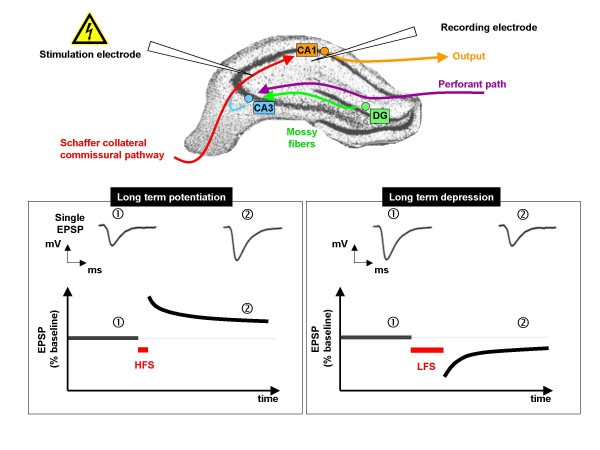
**Schematic representation of induction and recording of long-term potentation and long term depression in the hippocampus**. Long-term potentiation (LTP) and long-term depression (LTD) can be induced by applying an electrical stimulus by placing an electrode placed in the Schaffer collateral-commissural (SCC) pathway and recording from the CA1 subfield. *Upper panel shows *a coronal section through the dorsal hippocampus, with schematic representation of intra-hippocampal connectivity. The CA1 pyramidal cell layer receives input from the entorhinal cortex through the dentate gyrus [DG] and the CA3 pyramidal layers and the SCC; the subiculum carries hippocampal efferents. *Lower left-hand panel *illustrates measurements of LTP as excitatory postsynaptic potentials (EPSP, peak amplitude or slope of the latter). Initially, low-frequency stimulation (LFS, usually less than 0.1 Hz) is applied to the Schaffer collaterals to establish a stable baseline (usually for 20-30 min), after which LTP is induced by high-frequency stimulation (HFS; usually 100 Hz), followed by LFS. Successful induction of LTP can be assumed when the post-HFS EPSP peak amplitude (or slope) exceeds that seen before HFS and is maintained for at least 60 min. ① depicts a single evoked EPSP; ② represents a potentiated EPSP after HFS. *Lower right-hand panel *shows that EPSP recordings also serve to detect LTD. After initial baseline recording, low-frequency stimulation (LFS, usually 1 or 5 Hz) is applied to the SCC; successfully induced LTD can be assumed when the post-LFS EPSP peak amplitude (or slope) is smaller than that observed before LFS. ① shows a single baseline EPSP; ② depicts a example of a depressed EPSP after LFS.

Many of the changes in behavior and brain physiology that are listed in Additional File 1 reflect rapid responses of the hippocampus to steroid hormones. For example, corticosteroids have been consistently shown to influence cognition and their effects are thought to result from their ability to directly or indirectly alter the excitability of hippocampal neurons. The hippocampus has been extensively studied for a number of pragmatic reasons. The input-output connections of the different hippocampal subfields are well defined, making their electrophysiological study convenient. Of all brain areas, the hippocampus has been best studied in the context of long-term potentiation (LTP) and long-term depression (LTD), the electrophysiological correlates of learning and memory, functions in which the hippocampus is strongly implicated [[33-35]; see Figure 2 and Additional File 2]. The hippocampus also serves as an important homeostatic regulator of the HPA axis upon which it exerts a strong negative drive [36,37] through the mediation of nMR and nGR [38].

Although the attention paid to the hippocampus is justifiable because of its role in the regulation of many behavioral and physiological processes, it should be remembered that it constitutes only part of a complex neuronal network that underpins physiology and behavior in normal and pathological states. For example, although the hippocampus plays an important role in the regulation of the HPA axis, it should be noted that other brain areas such as the prefrontal cortex [39], amygdala and bed nucleus of the stria terminalis, under the modulatory influence of monoamines from the hindbrain [40], contribute to the control of corticosteroid secretion; all these areas have reciprocal connections with the hippocampus and express nGR.

Several studies have begun to define how corticosteroids and other steroids act on different brain structures to produce integrated and adaptive behavioral and physiological responses, e.g. the prefrontal and orbito-frontal cortices (executive functions, including attention, behavioral flexibility, declarative memory, decision making [41,13,14,42]), thalamus (processing and gating of sensory input [43], amygdala (evaluation of emotional load of sensory input and regulation of fear [44], ventral striatum (motivation and reward [45] and decision-making [42]), and the cerebellum (learning of motor tasks [46]. Of these, the amygdala, involved in the control of fear, aggression and cognition (see Additional File 1), has been the most intensively studied. Interesting work by Roozendaal and colleagues has demonstrated a cross-talk between rapid GC and noradrenergic signaling in contextual memory consolidation [44,47] and suggests that endocannabinoids are key mediators of this cross-talk [48].

## Putative membrane receptors - pirates with legs to stand on?

The message that emerges from the previous section is that nuclear receptors, acting as transcriptional factors, are unlikely to mediate rapid actions of the sort listed in Additional File 1. Nevertheless, the identity of the molecular entity that allows rapid transduction of steroid signals remains elusive. Interestingly, some of the fast responses to corticosteroids are reportedly attenuated in the presence of pharmacological antagonists of nGR (RU 38486 [49] or nMR (spironolactone [50,51]). These findings suggest certain homologies between the classical nuclear receptors and the putative receptors mediating the rapid actions of these steroids. Nevertheless, the existence of another class of receptors, with distinct chemistries and cellular localizations, and that are not sensitive to the above-named antagonists, cannot be dismissed.

**Figure 3:**
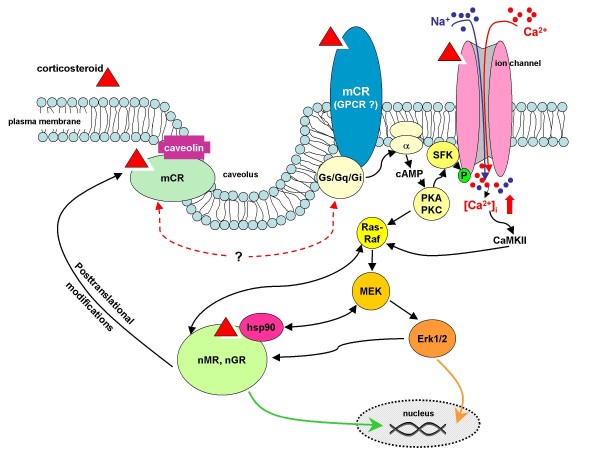
**Schematic representation of corticosteroid-triggered multiple tentative rapidly influencing neuronal function**. Corticosteroids are represented by red triangles. Nuclear GR (nGR) interact with caveolins [cf. [81]]; the interaction probably depends on posttranslational modifications of nGR to yield so-called membrane corticosteroid receptors (mCR). Alternatively, CS-initiated intracellular signaling cascades may result from corticosteroid binding to proteins embedded in the plasma membrane, e.g. G-protein-coupled receptors (GPCR) [75] which, upon activation, activate protein kinase A (PKA) and protein kinase C (PKC) in turn. Other evidence points to membrane-bound corticosteroid binding proteins that interact with members of the src family of kinases (SFK) to activate the mitogen-activated protein kinase (MAPK) pathway and/or modulate the activity of other membrane-associated proteins, e.g. NMDA receptors and other ion channels with potential steroid binding sites [76,85,86,222]. Under basal conditions, nGR are tethered in the cytoplasm in the form of a protein complex that includes the chaperone heat shock protein 90 (hsp90) which itself may directly interact with Src kinases and the MAPK kinase, MEK [cf. [83]]. Additionally, direct interactions between the nGR and Ras which may be functionally relevant have been described [84]. Finally, MAPK-mediated phsophorylation of nGR may influence the transcriptional activity of nGR [32]. Thus, corticosteroid actions at the plasma membrane can converge and prime or potentiate hormonal actions on gene transcription.

Several mechanisms that may account for membrane-mediated transduction of the rapid actions of estradiol have been proposed (see Figure 3). Substantial evidence supports the view that classical nuclear estrogen receptors (nER of which there are two isoforms, ERα and ERβ) are integrated into, or in close proximity of, the cell membrane. One hypothesis is that palmitoylation facilitates the interaction of these receptors with caveolins, a family of proteins that associate with cholesterol and sphingolipids to form caveolae within the plasma membrane and which are implicated in signal transduction. While some authors describe protein-protein interactions of such membrane-associated nER with other membrane proteins as a mechanism to explain rapid estrogen signaling [52-54], others propose mediation by a membrane-bound ER (mER) that is coupled to a Gα_q _protein. Evidence for the latter includes the observation that estradiol induces activation of the phospholipase C- protein kinase A (PLC-PKC-PKA) pathway in nER knockdown mice [55]. The same investigators demonstrated rapid electrophysiological effects of STX, a diphenylacrylamide-based selective estrogen receptor modulator, in nER knockout animals; STX, which does not bind to either isoform of the nER, proved to be more potent than estradiol in their *in vitro *and *in vivo *test systems [55,56].

While no mER has been cloned and characterized to date, GPR 30, an orphan G protein-coupled receptor (GPCR), has been identified as a potential transducer of estrogen signals that originate at the cell membrane [57,58]. GPR 30 was shown to display similar structural characteristics to other membrane receptors [57], but was nevertheless viewed with a certain amount of skepticism. For example, the nER antagonist ICI 182,780 exerts agonistic effects on this receptor [59] and neurons from GPR 30 knockout mice still display rapid responses to estradiol [60], the latter finding suggesting that GPR 30 may co-exist alongside (an)other mER with unique pharmacological properties. Notably, in an extension of their earlier work, Revankar et al. [61] exploited chemical biology to explore the subcellular localization of GPR 30 and its signaling potential; on the basis of observations in 4 cancer cell lines, they discarded the notion that sufficient GPR 30 is localized at the plasma membrane and rather suggested that GPR 30 localized in the endoplasmic reticulum serves as an intracellular transmembrane receptor for estrogen.

Interestingly, Toran-Allerand and colleagues [62,63] described a high affinity (K_D _for estradiol: 1.6 nM) caveolin-associated protein in the plasma membranes of neonatal (but not adult) neocortical and uterine tissues. This so-called ER-X seems to come closer to meeting the expectations of a distinct mER insofar that it cannot be blocked by ICI 182,780 [62]; moreover, these authors found that experimentally-induced ischemic stroke in adult animals is accompanied by an upregulation of ER-X in the brain, suggesting that the ER-X mediates the neuroprotective actions ascribed to estrogens.

It is tempting to hypothesize, that the mediators of rapid corticosteroid effects may share similar basic properties and mechanisms with the proposed membrane-associated estrogen receptors. The existence of a membrane-bound receptor for corticosteroids (herein referred to as mCR) was postulated by Willmer in 1961 [64]. Willmer's suggestion that steroid hormones interdigitate with, and alter the permeability of, lipids in the plasma membrane, lost currency as evidence that steroids bind to intracellular proteins (nuclear receptors) and stimulate protein synthesis began to accumulate from 1961 onwards [65,66]. However, in 1974 Satre and Vignais described corticosterone binding to mitochondrial preparations from the adrenal and kidney [67], a finding that eventually extended to other cell types [68]. A series of authors provided evidence for membrane-bound steroid recognition sites in the brain [69-71]; among these, Towle and Sze demonstrated specific corticosterone binding to plasma membrane preparations from rat brain synapses [72]. These membrane binding sites had a relatively high affinity for corticosterone (K_D _10^-7 ^M *vs*. 10^-9 ^M in the case of cytosolic binding sites) and treatment with phospholipase A2 or phospholipase C led to complete dissociation of membrane-bound corticosterone. Similarly, Orchinik et al. described the presence of mCR in brain synaptosomal fractions obtained from the amphibian *Taricha granulosa *(rough-skinned newt) [69]. These receptors showed pharmacological specificity for corticosterone and cortisol (K_D _10^-9 ^M), and lesser affinities for aldosterone and other natural and synthetic steroids (such as dexamethasone and RU 38486). Importantly, Orchinik et al. reported a linear relationship between the potencies of various compounds (corticosterone being the most potent) in inhibiting male reproductive behavior (inhibition by corticosterone within 8 minutes of application) and their ability to bind the putative mCR [69]. In subsequent studies, these authors described similar neuronal mCR in mammalian [73] and bird [74] brains and suggested a role for guanine nucleotide-binding proteins in the formation of a ternary complex of corticosterone and the putative neuronal mCR, i.e. the mCR appears to be coupled to G proteins [75]. Additional evidence for the existence of a mCR was eventually provided by Orchinik's colleagues who solubilized and partially purified membrane-bound corticosterone binding sites from the amphibian brain [76]; the assumed mCR had a molecular weight of about 63 kDa, as compared to 97 kDa and 110 kDa in the case of the nGR and nMR, respectively. More recently, studies by Johnson et al. [77] provided anatomical evidence for the existence of nGR within the postsynaptic density of neurons in the rodent amygdala. At present it is unclear as to whether there are any homologies between the mCR and either the nGR or nMR.

Ultrastructural studies with an antibody against purified rat nGR revealed immunoreactivity associated with the plasma membrane of rat hippocampal and hypothalamic neurons [78]. Notably, membrane-associated immunoreactive nGR sites were observed in or near membranes covering the dendrites and somata of pyramidal neurons; nGR immunoreactivity was also seen in the vicinity of the Golgi complex. With regard to the plasma membrane, Liposits and Bohn [78] noted that nGR immunoreactivity was associated with coated vesicles which, together with their localization along the membrane, suggested that nGR might either be transported and inserted into the plasma membrane, or coupled to mediators of transduced signals. In this respect, parallels may be drawn with what was reported above with respect to the membrane-bound mediators of estrogen actions. Palmitoylation of the nER has been suggested as a mechanism that facilitates integration of the nuclear receptor into (or the proximity of) the cell membrane, thus providing access to BSA-conjugated steroids and interactions of the receptor with membrane-associated signaling proteins [79]. While it remains to be shown that classical corticosteroid receptors can be palmitoylated and trafficked to the plasma membrane, recent studies have identified a highly conserved 9-amino acid motif in the ligand binding domain of estrogen, progesterone, androgen and glucocorticoid receptors that could serve as a substrate for palmitoylation [80]; these observations suggest that palmitoylation may be a general mechanism that allows nuclear receptors to double up as *bona fide *membrane receptors. Supporting the plausibility of this view, Matthews et al. have shown that nGR interacts with caveolin [81].

Many unliganded nuclear receptors (e.g. nGR), are tethered in the cytoplasm through their association with chaperone proteins such as heat shock protein 90 (hsp90); this complex is dissociated upon arrival of the ligand [24]. Interestingly, hsp90 is known to interact with src kinase [82], a membrane-proximal kinase thought to mediate the rapid activation of the MAPK pathway by corticosteroids. In addition, hsp90 interactions with MEK2, another kinase upstream of MAPK, has been shown to mediate MAPK pathway activation by estradiol [83]. In fact, nGR itself reportedly interacts with Raf-1, a downstream effector of Ras, and upstream regulator of the MAPK pathway [84].

Receptors for several neurotransmitters (some of which are ion channels) have been shown to bind CS [76,85,86]. Although it remains unclear as to whether these interactions serve as a conduit of the rapid actions of CS, the latter seems plausible given the evidence that neurosteroids can modulate chloride flux and thereby, neuronal excitability, by binding to an allosteric site on the GABA_A _receptor [87].

In summary, there is growing support for the view that CS can initiate signaling at the plasma membrane through one or more of the following mediatory mechanisms: (i) G protein-coupled membrane-bound CS receptors, (ii) steroid modulatory sites on plasma-bound neurotransmitter receptors, (iii) interactions between cytoplasmic CS receptors and kinase family-interacting chaperone molecules, and/or (iv) palmitoylation. Elucidation of the mechanisms underlying the rapid actions of CS will require a stepwise analysis of the contributions of each member of this 'interactome' - a major challenge.

## From the sightlines - peeping on a rapidly changing stage

This section will focus on the cellular endpoints that can be used to support the view that corticosteroids rapidly influence neuronal activity, focusing on alterations in membrane excitability and signaling cascades that originate at or close to the plasma membrane. However, attempts to summarize the existing literature are confronted with the fact that the results derive from disparate protocols and experimental models in different laboratories. For example, a wide range of corticosteroid doses and exposure times have been applied to studying synaptic transmission in either rat or mouse dissociated hippocampal neurons or hippocampal slices. We will, however, first consider early studies on hypothalamic neurons by Kasai and colleagues and Saphier and Feldman, using *in vitro *ionotophoresis. Kasai and colleagues showed that cortisol excited tuberoinfundibular neurons in the paraventricular nucleus (PVN) which project to the median eminence from where their neurosecretory products reach the anterior pituitary; however, these authors also reported inhibitory effects of cortisol in the PVN, suggesting this to result from inhibition of noradrenergic inputs [88-90]. Saphier and Feldman, observed a significant reduction in the spontaneous firing rates of similar hypothalamic neurons after the application of corticosterone [91,92]; these changes had a rapid onset and were maintained even after iontophoresis of the hormone was stopped. Further, they reported on a subset of neurons whose activity was not altered by corticosterone; glutamate-induced excitation of these neurons was however suppressed in the presence of corticosterone.

Together, the studies described above represent a hypothalamic electrophysiological correlate of the negative feedback control of adrenocortical secretion, and illustrate that corticosteroids can elicit different responses from different brain areas or neuronal populations within an anatomical region or specific neuronal phenotypes within a given subfield; moreover, the responses depend on neural inputs to the particular set of neurons under investigation [91,93]. Given the suggested importance of the hippocampus in mediating glucocorticoid negative feedback (see above), it is surprising that Barak [94] failed to observe any changes in the activity of hippocampal neurons upon applying corticosterone. As will become evident below, despite a large number of studies that focussed on the CA1 subfield of the hippocampus, it is difficult to compile a consensus view of how corticosteroids impact on the activity of this region.

Examining spike accommodation in hippocampal neurons, Vidal et al. reported that corticosterone (1 μM) decreases spike numbers [95], whereas Joëls and de Kloet [96] and Beck et al. [97], using 1 nM, observed the steroid to increase spike numbers and decrease the after-hyperpolarisation (AHP) amplitude; these effects were abolished in the presence of spironolactone (nMR antagonist). Importantly, 30 nM of corticosterone, which activates nGR (as well as nMR), decreased spike numbers and increased AHP amplitude, leading the authors to conclude that the bifurcating actions of low and high doses of corticosterone reflect the activation of nMR and nGR, respectively [96]. Further, given the gradual rise in corticosterone levels upon arrival of a stimulus (e.g. stress), they proposed a concentration-dependent biphasic cellular response to corticosterone, i.e. an initial increase in neuronal excitability, followed by suppression of neuronal excitability. Similar findings were reported earlier by Rey et al. (effects observed between 0.2 and 10 nM corticosterone; peak increase in spike amplitude at 2 nM corticosterone) [98].

Given that the amplitude of the AHP is determined by Ca^2+ ^and Ca^2+^-dependent K^+ ^transients [99,100], it is interesting that Landfield and colleagues reported that high doses of the synthetic GR agonist RU28362 (7 μM) enhance the amplitudes of voltage-dependent calcium channel (VDCC)- mediated Ca^2+ ^spikes in a protein synthesis-dependent manner [101]. In contrast, Tian et al. suggested that the increase in the slow after-hyperpolarization amplitude seen after exposure to high doses of corticosterone may involve cAMP-dependent phosphorylation and Ca^2+^-activated K^+ ^channels [102]: dexamethasone (1 μM), a synthetic glucocorticoid with high selectivity for the nGR, blocked PKA-mediated inhibition of Ca^2+^-activated K^+ ^channels without influencing VDCC-mediated Ca^2+ ^currents in a mouse pituitary cell line (AtT20). It should be noted that Tian et al. treated their cells with dexamethasone for 2 h and that these effects required *de novo *protein synthesis for their manifestation [102,103]. Because activation of NMDA receptors results in an influx of Ca^2+ ^and, as mentioned above, Ca^2+ ^determines the AHP amplitude [99], corticosteroid-NMDA receptor interactions have been analyzed in a number of studies using electrophysiological recordings as the endpoint. For example, Wiegert et al. showed that exposure of mouse hippocampal slices to corticosterone (100 nM) for 20 min resulted in NMDA receptor-mediated suppression of primed-burst potentiation and synaptic potentiation [104] (induced by stimulation at 10 Hz, in contrast to the more commonly-used 100 Hz LTP regimen). In contrast, theta-burst potentiation (see Additional File 2 for information on different stimulation protocols), which requires activation of both NMDA receptors and voltage-dependent Ca^2+^-channels was not affected by corticosterone treatment. The same authors also described a role for L-type Ca^2+ ^channels in the synaptic actions of corticosterone [105]. In the context of the question of whether corticosterone can rapidly alter synaptic function, it is important to note, however, that Wiegert et al. [104] and Chameau et al. [105] made their electrophysiological recordings between 1 and 6 h after initial exposure to the steroid. On the other hand, Chameau et al. [105] found by quantitative PCR that corticosterone did not change the mRNA expression of the pore-forming Ca_v_1 subunit of the L-type Ca^2+ ^channel, and ruled out transcriptional mechanisms in the effects they observed.

Wiegert et al. [104] showed that RU 38486 blocks corticosterone-induced impairments of synaptic plasticity, implying mediation of the effects by nGR. A similar conclusion was drawn from their previous work on GR^dim/dim ^mice, a strain carrying a point mutation of the DNA binding domain of the nGR which precludes transcriptional effects; briefly corticosterone did not influence VDCC-mediated Ca^2+ ^currents in hippocampal slices from GR^dim/dim ^mice [106]. To address the question of how glucocorticoids enhance Ca^2+ ^currents on the one hand, and reduce synaptic efficacy on the other, Joëls' laboratory examined synaptic efficacy 1-4 h after a brief exposure to corticosterone (1 μM CORT for 20 min) [107]. Their investigations revealed that synaptic transmission was potentiated when VDCCs were activated, and impaired only when NMDA receptors were activated; moreover, they found that these effects were RU 38486-sensitive, indicating their mediation by nGR. Together, these observations point to the importance of considering all of the individual components that contribute to the overall response in field recordings. In this respect, it is worth recalling that the magnitude of LTP and LTD is a function of the number of AMPA receptors that are present at the synaptic surface (see Additional File 2). Miniature excitatory postsynaptic currents (mEPSCs, which represent the spontaneous release of neurotransmitter quanta from presynaptic terminals) are mediated by AMPA receptors and changes in the mEPSC amplitude represent postsynaptic changes in AMPA receptor properties and/or numbers. Indeed, Martin et al. observed that corticosterone increases the amplitude (but not frequency) of miniature excitatory postsynaptic currents and demonstrated that corticosterone increases trafficking of the GluR1 and GluR2 subunits of the AMPA receptor to the synaptic surface, apparently through an nGR-dependent mechanism [108]. This last study is in good agreement with that by Karst and Joëls, who also reported nGR-mediated increases in mEPSC amplitude [109].

Despite the overwhelming amount of data implying a role for nGR and/or nMR in mediating the effects of corticosterone on synaptic transmission, other evidence indicates that the rapid actions of corticosterone are mediated by mCR. For example, corticosterone was shown to dose-dependently (0.1, 1, 10, 100 μM) inhibit inward NMDA receptor-mediated currents, within seconds, in primary hippocampal cultures [110]. This effect faded upon wash-out of the hormone and was not reversible with RU 38486; assuming that RU 38486 binds specifically to nGR, the latter finding precludes mediation through nGR. The latter interpretation is supported by the finding that the effects of corticosterone were reproducible with membrane-impermeable BSA-conjugated corticosterone. Results from Takahashi et al. also dismissed a mediatory role for nGR or nMR in the mediation of corticosterone effects; however, they reported that the steroid prolongs the elevation of NMDAR-mediated Ca^2+ ^influx in dissociated hippocampal neurons independently of VDCC and mobilization of intracellular Ca^2+ ^stores [111]. In contrast, other authors reported that corticosterone and BSA-corticosterone (30 min) inhibit the peak amplitude of NMDA receptor-mediated Ca^2+ ^currents in the CA1 subfield of the mouse hippocampus [93], that bath application of corticosterone to hippocampal slices inhibits VDCC-mediated Ca^2+ ^currents within minutes [112], and that corticosterone increases synaptosomal uptake of Ca^2+ ^upon K^+^-induced depolarization [113].

At this stage, it is important to note that some of the discrepant reports on corticosterone-induced changes in NMDAR-mediated Ca^2+ ^currents may reflect the different durations of exposure to the steroid used by different groups. In fact, Wiegert et al. defined a narrow time window (10 min before high frequency stimulation) during which corticosterone facilitates synaptic potentiation; longer bath applications of the hormone were found to impair synaptic potentiation [114].

Most of the evidence reviewed above presumes post-synaptic sites of corticosterone action. New studies of CA1 neurons also report changes in the frequency of mEPSCs, thus implying presynaptic sites of action. Thus, Karst et al. [50] and Olijslagers et al. [51] showed that corticosterone increases the frequency of AMPA receptor-mediated mEPSCs. Both studies show that application of BSA-conjugated corticosterone produced similar effects to those obtained with corticosterone, and interestingly, that *de novo *protein synthesis was not essential for their manifestation. Together, these results hint at the involvement of receptors other than nGR and nMR; nevertheless, nMR antagonism by spironolactone resulted in a blockade of the corticosterone-induced increases in mEPSC frequency. [50,51] [but see [114]]. On the other hand, since RU 28362, a synthetic nGR agonist, did not reproduce the effects of corticosterone, and because the effects were not antagonizable with RU 38486, Karst et al. [50] and Olijslagers et al. [51] proposed that the putative mCR might share identity with the nMR. The latter suggestion is supported by experiments in mice with targeted mutations of nGR and nMR [50,106] and work by Groc et al. [115]. Using dissociated hippocampal cells to visualize AMPA receptor trafficking, the latter authors observed increased synaptic surface expression of GluR2 subunits of the AMPA receptor within minutes of exposure to corticosterone, BSA-conjugated corticosterone or aldosterone (the prototypic nMR agonist).

Related to the electrophysiological measures summarized in the last few paragraphs, Olijslagers et al. demonstrated that activation of the MAP kinase ERK1/2 is crucial for the corticosterone-induced increase in mEPSC frequency [51]. Interestingly, their experiments showed non-dependence on postsynaptic G protein activity on mEPSC frequency. Rather, by using the H-Ras G12V strain of mouse which displays strong presynaptic activation of ERK1/2 due to constitutively high expression of the H-Ras transgene, they suggested that the actions of corticosterone are initiated at presynaptic sites, increasing the probability of presynaptic neurotransmitter release [50,51]. Moreover, in agreement with other studies [111], Olijslagers et al., reported that intracellular Ca^2+ ^stores do not influence mEPSC frequency upon exposure to corticosterone [51]. Lastly, it should be noted that although the involvement of G proteins in corticosterone-induced changes in mEPSC frequency were excluded [51], direct infusion of GDPβS into the postsynaptic cell prevented the decrease of the peak amplitude of I_A_currents (postsynaptic K^+ ^conductance) by corticosterone [51]; this finding points to mediation through a postsynaptic mCR-dependent mechanism.

A number of studies suggest a role of G proteins in the mediation of the rapid actions of corticosterone. For example, ffrench-Mullen showed that the inhibition of Ca^2+ ^currents by cortisol in guinea pig CA1 neurons depends on pertussis toxin-sensitive G-proteins [112]. The same author also showed that the effects of cortisol are significantly diminished in the presence of PKC inhibitors (BIS and PKCI 19-31), and ruled out a role for PKA in the mediation of the actions of cortisol [112]. Similarly, Chen and Qiu showed that corticosterone rapidly inhibits VDCC-mediated Ca^2+ ^currents in a phaeochromocytoma cell line of neural origin (PC12 cells), and that inhibition of G proteins by application of either pertussis toxin or GDPβS significantly attenuates the ability of either corticosterone or BSA-corticosterone to stimulate the influx of Ca^2+ ^[116]. They also demonstrated that activation of PKC with phorbol 12-myristate 13-acetate results in an inhibition of Ca^2+ ^entry though VDCC after depolarization with K^+^, and that the application of corticosterone activates PKC within 5-15 minutes. Lastly, like Qi et al. [117] who obtained similar results in primary hippocampal neurons, Chen and Qiu [116] showed that both, corticosterone and BSA-conjugated corticosterone trigger the activation of PKC and a series of MAP kinases (ERK1/2, p38MAPK and c-Jun) in PC-12 cells; maximum kinase activation occurred within 15 min of application of the hormone and the effects could not be attenuated by RU 38486.

## Reality

Blood (and brain) corticosteroid levels rise and fall in a pulsatile manner under basal (unstimulated) conditions, and the circadian and stress-induced rises in corticosterone secretion occur gradually, taking minutes or even hours to reach peak levels. This raises the question of whether corticosteroid levels above a certain threshold have an impact on physiology and behavior and provokes curiosity about the mechanisms that could underpin the rapid biological actions of corticosteroids. Original interest in the fast actions of corticosteroids was awakened by attempts to understand the 'fast' and 'slow' negative feedback actions of corticosteroids at the level of the pituitary and the brain. Pioneering research by Mary Dallman used ingenious experimental designs which eventually provided evidence for the rapid actions of corticosteroids in reducing their own secretion [118] and, as already mentioned, the search for electrophysiological correlates was pursued in the hypothalamus in parallel. Today, predominantly based on work from the laboratories of Stafford Lightman and colleagues [4], it would appear that the ultradian rhythmic secretion of relatively high-amplitude corticosterone may serve to ensure low levels of adrenocortical activity during the organism's resting phases; these brief pulses presumably act rapidly to suppress brain-pituitary drive of adrenal secretion.

At the behavioral level, Orchinik et al. [69] elegantly demonstrated the potency of corticosterone in inhibiting male reproductive behaviour in newts, within 8 min of application. In mammals, Jozsef Haller and colleagues have shown that corticosterone injections elicit aggressive and anxiety-related behavior (latency of 7 min) in rats whose endogenous adrenocortical activity is suppressed by inhibition of 11β-hydroxylase activity with metyrapone [119-121]. Several authors have also described the ability of corticosterone to rapidly alter locomotor behavior in rodents; for example, acute systemic injections of corticosterone to rats (placed in a novel environment) were shown to stimulate locomotion within 7.5 minutes of administration [122].

Rhythms in the secretion of corticosteroids and other neuromodulatory molecules can influence experimental outcomes, even in *in vitro *settings. For instance, Ca^2+ ^currents into hippocampal CA3 neurons in *in vitro *preparations are highest during the subjective night, when corticosterone levels are highest [123]. Similarly, Brunel and de Montigny [124] reported that the firing rate and pharmacological responsiveness of CA3 neurons is highest during the nocturnal peak in corticosterone secretion *in vivo*. Importantly, using hippocampal slice cultures, Chaudhury et al. demonstrated that the amplitude of LTP is greatest during the subjective night [125]. Additionally, Eckel-Mahan and colleagues reported circadian dependency in the efficiency of consolidation of long term memory [126].

Many studies support the idea that stress, a large part of whose actions are mediated by corticosteroids, influences learning and memory. Besides the quality and intensity of the stressor, the context in which the stressful stimulus is perceived, is an important determinant of the behavioral outcome. The latter is more easily explained in terms of 'intrinsic' and 'extrinsic stress [127]; 'intrinsic stress' refers to situations in which stress is either elicited by, or directly associated with, the cognitive experience (e.g. spatial learning), whereas 'extrinsic stress' describes situations in which the stress occurs outside the context of the momentary stress situation (e.g. foot shock stress *before *spatial learning). According to a model developed by Sandi and Pinelo-Nava [127], learning and memory will be facilitated by stressors that activate the same (or similar) neural circuitries that are required for interpreting and responding to a particular cognitive challenge. Supporting this view, Cahill and McGaugh [128] and Sandi [129] reported that emotionally arousing experiences are better remembered than neutral ones. In fear conditioning experiments, Cordero et al. noted that post-training corticosterone levels correlate with the strength of stimulus required to encode memories [130,131]. Moreover, the importance of corticosterone in information acquisition and consolidation of memory is well known, even if still poorly understood [132-135]. The relative importance of nMR and nGR in these processes are elegantly discussed by Schwabe et al. [136], and Revest et al. [134] have demonstrated a mediatory role of the MAPK pathway in the facilitation of hippocampus-dependent contextual fear conditioning by corticosteroids. In the previously-cited work on long-term contextual fear memory by Eckel-Mahan and colleagues [126], rhythms of MAPK (ERK1/2) activation were shown to coincide temporally with the degree of persistence of memory. Given that corticosterone acutely increases ERK1/2 phosphorylation [51,116,117,134], the results presented by Eckel-Mahan and colleagues [126] should be considered in the context of the hypothesis proposed by Sandi and Pinelo-Nava [127] and the pioneering work by Oitzl and de Kloet [137]; in addition, since the amygdala plays a major part in the regulation of fear and has reciprocal interactions with the hippocampus and other cognition-regulating brain areas, future interpretations of the work by Eckel-Mahan and colleagues [126] should embrace the idea that corticosteroids can exert actions on a network of interconnected brain structures, whose individual responses will determine the ultimate behavioral output.

Besides the acute behavioral and physiological actions of corticosteroids, much research has been focused on understanding the influence of chronically elevated corticosteroid secretion. Notwithstanding the above-mentioned fact that corticosteroids may exert acute effects during the rising phase of the endocrine response to stress, it is important to note that the latter is, generally, a protracted one. Thus, while the acute rises in corticosteroid secretion may shape the overall long-term response, the longer duration of corticosteroid exposure after stress allows recruitment of an array of intracellular responses (including nuclear receptor-mediated events) and cellular, physiological and behavioral adaptations. It is important to note that, although the adrenocortical response to stress primarily serves an adaptive purpose, in certain circumstances, it may switch to being maladaptive, marked by transient or chronic pathology, as discussed earlier in this article.

The physiological and behavioral responses to stress depend on myriad molecules and processes, with an important contribution by corticosteroids; effects of the latter are often studied in isolation at the cost of other contributory factors and the neural networks which regulate, or may be regulated by, corticosteroids. This can be exemplified by considering our earlier discussion of corticosteroid interactions with glutamatergic transmission and reports that the direction and/or magnitude of LTP and LTD are influenced by the intensity and emotional value of a given stressor; for example, LTP is only reduced in animals exposed to uncontrollable stress [138], but not in animals that can escape from the stressor [139]. Using the paradigm of foot-shock stress, Wang et al. reported that stress induces a shift in synaptic plasticity; thus, whereas stress facilitates LTD induction, it impairs LTP induction [140]. Besides showing that these effects of stress can be blocked by RU 38486, these last authors showed that blockade of the NMDA receptor restores LTP inducibility in stressed animals; further they demonstrated that stress-induced changes in synaptic efficacy can be abolished by prior administration of Ro25-6981, a specific antagonist of the NR2B subunit of the NMDA receptor. A role for the NR2B subunit in the synaptic plasticity thought to be essential for the orchestration of the behavioral response to stress was also suggested by Wong et al. who showed that Ro25-6981 reverses elevated platform stress-induced deficits in spatial learning and memory, as tested in the Morris water maze (MWM) [141].

The NR2B subunit is predominantly associated with extrasynaptic NMDA receptors whose activation depends on glutamate "spill-over", a phenomenon that can be mimicked with *threo*-β-benzyloxyaspartate (TBOA), a blocker of glutamate re-uptake. Wong et al. [141] found that TBOA application to animals 5 min before low frequency stimulation resulted in the successful induction of LTD, indicating that stress leads to glutamate "spill-over". Linking LTD with stress-induced memory impairment, the authors showed that preventing LTD induction by infusion of a GluR2 peptide analogue that cannot be internalized abolished the ability of stress to cause memory deficits in the MWM test; these findings add to the evidence that acute stress results in the internalization of AMPA receptors, followed by synaptic depression and learning and memory deficits.

We previously discussed how the MAPK signaling pathways may be linked with LTP and LTD (and learning and memory). In this respect, it is interesting to note that this pathway is concomitantly activated by stress, presumably due to activation of nGR [142,143], believed to be essential for the phosphorylation of ERK1/2 [134]. Moreover, the observation that tail shock and restraint stress robustly activate ERK1/2 and impair synaptic potentiation in the CA1 subfield suggests a major role for the MAPK pathway in mediating the actions of stress [144]. In addition to inducing the phosphorylation of ERK1/2, stress activates other kinases (e.g. p38 MAPK, CaMKII) and pCREB within 2 min of swim stress [145]. Surprisingly, however, the latter responses are accompanied by a reinforcement (rather than impairment) of LTP in the dentate gyrus of the hippocampus. This finding indicates that different stressors may elicit quite different electrophysiological responses and/or, that the synaptic effects of stress differ from one hippocampal subfield to another. Since the effects of stress on biochemical and electrophysiological signalling in the dentate gyrus were found to be subject to modulation by serotonin [145], it is plausible that differential monoaminergic innervation of the different hippocampal subfields defines the ultimate cellular response.

**Figure 4:**
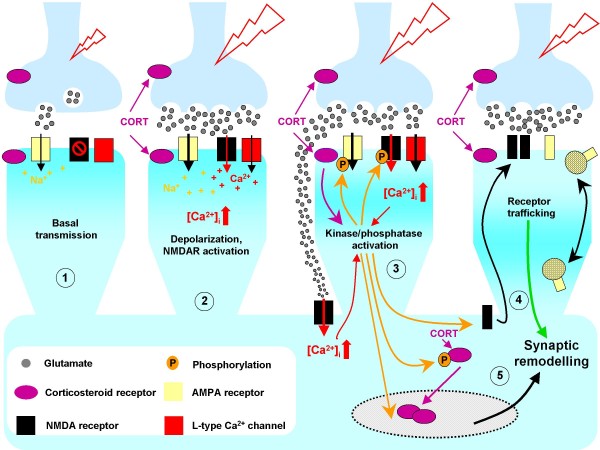
**Working model of sequential corticosteroid influences on synaptic physiology**. Corticosterone-mediated changes in synaptic transmission occur at different levels and in different sequential steps. ① depicts synaptic transmission under basal conditions. Neuronal excitation results in glutamate secretion from synaptic vesicles at presynaptic sites into the synaptic cleft. Glutamate binds to postsynaptic glutamate-gated ion channels (in particular, AMPA receptors), which open to permit ion fluxes (Na^+ ^influx, K^+ ^efflux) across the AMPA receptor, resulting in a depolarization of the postsynaptic cell. Due to a voltage-dependent Mg^2+ ^block in its membrane domain, the NMDA receptor remains inactive under basal conditions, and is activated when a certain transmission threshold is reached. ② Exposure to corticosteroids (e.g. during stress) may lead to activation of ERK1/2 in the presynaptic terminal (possibly through membrane corticosteroid receptors [51]); increased glutamatergic stimulation of postsynaptic AMPA receptors results in an increase in the frequency of AMPA receptor-mediated miniature postsynaptic currents (mEPSCs). ③ Enhanced activation of AMPA receptors in the previous step further depolarizes the postsynaptic membrane and activates NMDA receptors. Activated NMDA receptors (Na^+ ^and Ca^2+ ^influx, K^+ ^efflux) lead to further depolarization of the postsynaptic cell, resulting in the opening of voltage-dependent Ca^2+ ^channels (VDCC) and high postsynaptic concentrations of Ca^2+^. Corticosteroids may stimulate glutamate secretion so strongly, causing glutamate "spill-over" which activates not only synaptic, but also extrasynaptic, glutamate receptors [141]; the latter are mainly NMDA receptors of the NR2B subtype. The increased intracellular levels of Ca^2+ ^trigger a cascade of Ca^2+^-dependent signaling pathways in the postsynaptic cell, which may, in turn, induce the phosphorylation and de-phosphorylation of postsynaptic glutamatergic receptors and of nuclear corticosteroid receptors (nMR and nGR). Activation of extrasynaptic NMDA receptors is thought to trigger NR2B-dependent kinases, which might initiate trafficking of extrasynaptic NR2B receptors into the postsynaptic surface. Furthermore, Ca^2+^-dependent signaling pathways in the postsynaptic cell participate in the regulation of AMPA receptor trafficking to and from the synaptic surface, as indicated in ④. Phosphorylation of nuclear corticosteroid receptors, influences their translocation to the nucleus and therefore, their transcriptional activity [32], as indicated in ⑤.

We summarize some potential mechanisms that may account for the rapid and slower effects of corticosteroids on neuronal physiology, with a focus on synaptic events, in Figure 4. An attempt is made to show how signals originating at the neuronal surface are integrated both at the synaptic and transcriptional levels.

## Critique

From the preceding, it appears safe to assume that, irrespective of the behavioural or physiological outcomes, acute and chronic elevations of corticosteroid secretion initiate common mechanisms and biochemical processes; convergence of these events will depend on parameters such as exposure dosage and time, as well as the context in which they occur. Given the potential for convergence (as well as potentiation), improved knowledge of the initial stages of corticosteroid signalling, whether membrane- or nuclear receptor-mediated, is clearly desirable. Studies on the rapid neural actions of corticosteroids are likely to gain further interest, especially as newer analytical tools become available and knowledge about the fast actions of other steroid hormones grows. It therefore seems appropriate to list some critical issues and needs, the consideration of which may foster progress through cautious reflection:

• **definition of the terms "rapid" or "fast" actions **of corticosteroids in terms of the timeframe within which a clearly defined (electro)physiological, biochemical and/or behavioural response is elicited in animals or neuronal cell and brain slice preparations;

• standardized test protocols (**steroid dose, animal or cellular models, and sex**^c^**of animals**); in *in vitro *studies, **drug diffusion times and active concentrations **achieved at target cells should be controlled; similarly, in *in vivo *research, **pharmacokinetic factors**, including solvent and route of administration, should be considered; **age **of animals, but also of material used for *in vitro *testing, is important because of dynamic age-related changes in the expression of key partners such as glutamate receptor subunits [146]; since corticosteroids are secreted according to a strict **circadian **rhythm, both the availability of endogenous corticosteroids as well as of primary and secondary downstream effectors will vary over the day - this demands **testing at a given circadian time **to ensure comparable measurements [123-125].

• while **surgical adrenalectomy **is a useful approach to ensure that only the actions of exogenously-administered steroids are being recorded, the operation requires anaesthesia and may involve potentially confounding post-operative pain; **chemical adrenalectomy **is a good alternative (e.g. blockade of corticosteroid synthesis with metyrapone), but it may have (indirect) non-selective effects on the production of other steroids; adrenalectomy, in general, induces massive apoptosis and stimulates neurogenesis in the dentate gyrus within just a few hours, changes that probably result in **reorganized neuronal circuits and measurable outputs **[147].

• attention to the fact that **acute and chronic corticosteroid exposures **differ significantly, and that **administration of corticosteroids only mimics an intermediate phase of the organism's response to stress**;

• clear **exclusion of transcriptional and translational events **initiated by activation of cognate nuclear receptors;

## The show must (will) go on

While the nuclear receptor-mediated actions of corticosteroids are well established, those that appear to be mediated through non-classical, possibly membrane-bound receptors, have perhaps not received sufficient appreciation. The lack of consistent results (see need for standardization in previous section), compounded by the relatively fruitless hunt for putative membrane receptors, accounts for the scepticism that haunts this area of research. Increased respectability might be gained by initially seeking answers to some of the following questions:

• How can the neural actions ascribed to peripherally-produced corticosteroids be distinguished from those that result from those elicited by corticosteroids thought to be produced in neural tissue?

• Can the rapid actions of corticosteroids observed predominantly in the CA1 subfield of the hippocampus be generalized to other hippocampal subfields, or indeed other brain regions?

• Do the endpoints assessed after application of corticosteroids reflect actions exclusively at the hippocampus? *In vitro*, do we get only a partial (or perhaps, false) picture? *In vivo*, are we monitoring responses from a network of corticosteroid-sensitive brain regions? How are the outputs modulated by other neurochemical states and inputs?

• Do corticosteroids directly interact with membrane proteins? What is the chemical identity of these molecules? Are they distinct from the known nuclear receptors and if not,

◦ Do they represent post-translational modifications (e.g. palmitoylated versions of the nuclear receptors, as suggested for the mER)?

◦ Is there biochemical evidence for interactions with other known membrane receptors (e.g. glutamate receptors); do these receptors have allosteric binding sites for corticosteroids as well as for pharmacological antagonists of nMR and nGR? (cf. estrogens, progestins)

• How do events that are triggered by corticosteroids at the membrane funnel into long-term cellular and organismic adaptations (e.g. by positive or negative priming of the gene machinery regulated by nMR and nGR)?

• How do the rapid actions of corticosteroids contribute to their longer-lasting actions (e.g. 'priming' of nuclear receptor-mediated events?)

• Is it possible to define corticosteroid actions - fast and slow - in terms of spatio-temporal maps, keeping in mind that damage induced in a relatively short time in one area may take longer to spread to other interconnected areas [cf. [13]]?

• Is it feasible to generate genetic or pharmacological tools that will facilitate acceptance and further study of mCR?

## Appendix

a) Corticosteroids: way upstream - the title of this article is adapted from Alan Ayckbourne's stage play *Way Upstream *in which two couples on a boating holiday run into some strange happenings.

b) A painted cloth in front of which a short scene is played while the main stage set is changed.

c) Research on the rapid actions of corticosteroids has mainly exploited male rodents or tissues derived from them. Corticosteroid secretion is strongly influenced by sex, as are physiology and behaviour. Many of the physiological and behavioural readouts monitored in such studies reflect the prevailing sex steroid *milieu*; in females, sex steroids are secreted in a cyclical fashion.

## Competing interests

The authors declare that they have no competing interests.

## Authors' contributions

TR, AP and OFX wrote the manuscript; KC critically reviewed the manuscript and suggested improvements. All authors read and approved the final form of the manuscript.

## Supplementary Material

Additional file 1 **Summary of rapid effects of corticosteroids and estrogens on the central nervous system **[148-181]. Click here for file

Additional file 2 **Synaptic plasticity and learning and memory **[182-221]. Click here for file
