# Supplementary material for: Corticosteroids: way upstream
Source: Mol Brain. 2010 Jan 11;3:2. doi: 10.1186/1756-6606-3-2 (PMC2841592; doi:10.1186/1756-6606-3-2)
Supplement: Additional file 1 — Summary of rapid effects of corticosteroids and estrogens on the central nervous system [148-181]. [file 1756-6606-3-2-S1.PDF]

Summary of rapid effects of corticosteroids and estrogens on the central nervous system

**Corticosteroids**				
**Parameter**	**Effect [refs]**	**Latency (min)**	**Mechanistic insights**	**Other comments**
**Locomotion**	Increased locomotion in novel environment **[122, 148]**	7	- protein synthesis- independent<br>- nitric oxide-mediated	no effect in familiar environment
**Aggressive behavior**	Increased aggression in resident-intruder paradigm **[119, 120, 149]**	2-7	protein synthesis-independent	
**Anxiety, risk-taking behavior**	Increased risk assessment, but no anxiety-related behavior **[121]**	2	protein synthesis-independent	
**Learning and memory**	Impaired retrieval of declarative long-term memory **[150]**	< 60		humans; oral dose of cortisone
	Impaired memory in water maze task **[151, 152]**	30	insensitive to transcriptional inhibition **[152]**	
	Impaired retrieval contextual memory **[153]**	30		interactions with noradrenergic signaling
	Enhanced taste conditioning (learning) **[154, 155]**	< 20		
**Neuro-chemical**	Increased extracellular dopamine levels in nucleus accumbens (reward-motivational pathway) **[156]**	20		
	Increased NMDA-mediated firing of dopaminergic neurons in ventral tegmental area (reward-motivational pathway) **[49]**	15	blocked by nGR antagonist, RU38486	aldosterone has opposite effect
	Increased spike frequency in locus coeruleus neurons **[157]**	0.5		
	Inhibition of catecholamine uptake **[158]**		directly inhibits monoamine extraneuronal transporter	
	Increased p38 and JNK and ERK1/2 phosphorylation	15	-not blocked by nGR antagonist	

	in PC12 cell line **[159, 160]**		RU38486<br>- effects achieved with corticosterone-BSA conjugate	
**Neuro-endocrine regulation**	Triggers fast glucocorticoid negative feedback **[161]**	2		
	Inhibits neuron firing rate in hypothalamic paraventricular nucleus (PVN) **[89, 91, 162]**	<5		
	Reduces excitatory glutamatergic input to PVN, increases endocannabinoid release **[163, 164]**	3.5	- G-protein coupled receptor and retrograde endocannabinoid signaling	Dexamethasone by intracellular delivery

**Estrogens** [see **165]**				
**Parameter**	**Effect (refs)**	**Latency (min)**	**Mechanistic insights**	**Other comments**
**Synaptic plasticity in hippocampus**	Increased CA1 field response **[166]**	10		
	Augmentation of LTP **[167]**	3	Enhancement of NMDAR-mediated EPSPs	
	Suppression of LTD **[168]**	30	Effect of estrogen is greater in aged animals	
**Feeding behavior**	Attenuation of feeding **[55, 169]**		- activation of PKC and PKA pathways;<br>- desensitization of anorexigenic proopio-melanocortin neurons in arcuate nucleus<br>- orexigenic neuropeptide Y expression downregulated	selective activation of mER, using STX

**Gonado-tropin-releasing hormone (GnRH) secretion**	Induction of transcriptional factor CREB in GnRH neurons in hypo-thalamic (AVPV) neurons **[170]**	<30		
	Rapid changes in AVPV neuron firing rate **[171]**	15	- Modulation of noradrenergic inputs and SK channel function	
	1. Hyperpolarization of GnRH neurons **[172, 173]**<br>2. Increased GnRH-neuron firing rate **[174, 175]**	5<br><10	1. Pertussis-sensitive GPCR<br>2. Ca^{2+} fluxes	
	Norepinephrine-induced induction of cAMP increased in GT1-7 cell line **[176]**	15	Estradiol-BSA mimics the effect	effects persisted for 48h
**Sexual behavior**	Facilitation of female sexual receptivity **[177, 178]**	15	- Activation of PKA and PKC<br>- Protein-protein interaction between mERα and mGluR1	
**Spinal cord**	Spinal reflex (pain) **[179]**	10	nERα and nERβ activate a cdk5/ERK cascade, eventually phosphorylating NR2B subunit of NMDAR	
**Neuro-chemical**	Increased ERK1/2 phosphorylation in primary neuronal cultures **[180]**	2-10	Effect also achieved with estradiol-BSA	Similar effects with ICI 182,780 (nER antagonist)
	Increased dopamine efflux mediated via PKC and MAPK pathway activation in PC12 cell line **[181]**	9	- Direct interactions between ERα, ERβ and dopamine transporter (DAT)<br>- increased and decreased membraneous ERα and ERβ, respectively	
	MAPK pathway activated in neocortical explants from nERα knockout mice **[62]**		Membrane-associated, high affinity for estradiol	ER-X induced after ischemic stroke
