# Supplementary material for: Corticosteroids: way upstream
Source: Mol Brain. 2010 Jan 11;3:2. doi: 10.1186/1756-6606-3-2 (PMC2841592; doi:10.1186/1756-6606-3-2)
Supplement: Additional file 2 — Synaptic plasticity and learning and memory [182-221]. [file 1756-6606-3-2-S2.PDF]

Synaptic plasticity and learning and memory

Until the end of the 19th century, it was believed that brain networks were permanently fixed and that once the brain was hard-wired it could not be changed. The notion that the brain could undergo plastic changes and that changes at neural junctions might account for memory started to emerge with the philosopher Alexander Bain's suggestion in 1872 that *"Actions, sensations, and states of feeling, occurring together, or in close succession, tend to grow together, or cohere, in such a way that when any of them is afterwards presented to the mind, the others are apt to be brought up in idea"*. Later Eugenio Tanzi (in 1893) and Ramon y Cajal (in 1894) independently suggested that learning did not depend on the generation of new neurons but rather on plastic changes at "synapses", a term introduced by Foster and Sherrington (1897) to describe the "junctions between neurons". Tanzi and Cajal proposed that those plastic changes strengthened existing synapses, thereby improving information processing between two cells. Some 50 years later, in 1949, Donald Hebb suggested that cells might grow new connections to enhance their ability to communicate if appropriately stimulated. Hebb's rule states "*When an axon of cell* A *is near enough to excite a cell* B *and repeatedly or persistently takes part in firing it, some growth process or metabolic change takes place in one or both cells such that* A's *efficiency, as one of the cells firing* B, *is increased.*"

Long-term potentiation

Two phenomena, long-term potentiation (LTP) and long-term depression (LTD), considered to reflect synaptic plasticity and to be the electrophysiological correlates of learning and memory, were discovered by Terje Lomo when studying the connections between the perforant pathway and the dentate gyrus in the rabbit hippocampus **[182]**. He observed a potentiation of excitatory postsynaptic potentials (EPSP) in cells of the dentate gyrus after a train of high-frequency stimuli. LTP was fully characterized in the rabbit hippocampus in 1973 and has since been observed to occur in many brain areas, including the prefrontal cortex, cerebellum, amygdala, hippocampus, visual cortex and perirhinal cortex.

Studies of synaptic plasticity in the hippocampus generally involve low frequency stimulation (< 0.1 Hz) of the Schaffer collateral commisural (SCC) pathway to elicit baseline synaptic responses. LTP is then induced by delivering a brief pulse of *high-frequency stimulation* (HFS; usually a series of 50-100 stimuli at a high frequency of 100 or more Hz [usually 100 Hz for 1 s, tetanus]). Variations of this are: *theta-burst stimulation* which induces LTP by stimulating at 100 Hz (several bursts of 4 shocks spaced at intervals of 200 ms), and *primed-burst stimulation* in which LTP is induced by a single priming stimulus (1-50 Hz), followed at 200 ms by a single burst of 4 shocks at 100 Hz. These protocols, most commonly used on slice or cell preparations *in vitro*, are based on parameters observed in the rat hippocampus *in vivo*, during learning **[183]**. LTP is considered to have been successfully induced if a persistent increase in the amplitude (and slope of the rising phase) of the excitatory postsynaptic response (EPSP) that follows low-frequency stimulation occurs, and if the increased EPSP amplitude is maintained for at least 60 minutes after its induction.

At least *in vivo*, LTP persists for days to months **[184]**. Following HFS, LTP occurs in three steps: 1) post-tetanic potentiation (PTP), which occurs independently of NMDAR activation and is very short-lasting; 2) short-term potentiation (STP) or “early LTP” (eLTP), a form of LTP usually lasting <60 minutes; and 3) LTP or “late LTP” itself, where the potentiation is of a longer duration. Both STP and LTP are dependent on NMDA receptor activation but may be modulated by kinases (e.g. calcium-calmodulin dependent protein kinase, CaMKII) activity **[185]**.

Pharmacological experiments indicate that the evoked EPSP during LFS is only composed of AMPA receptor-mediated potentials and that NMDA receptors do not contribute to EPSPs **[186, 187, 188]**. Thus, AMPA receptors provide practically all the input-output function of a neuron during low-frequency synaptic transmission under physiological conditions. During high-frequency transmission, these AMPA receptor-mediated EPSPs summate and activate NMDA receptors, thus facilitating the induction of LTP. The increase in EPSP after HFS is due to an improvement of synaptic efficacy at the post- and presynaptic site. Enhanced neurotransmitter availability and retrograde

signalling at the presynaptic site, as well as postsynaptic alterations such as changes in postsynaptic AMPA receptor properties (phosphorylation/de-phosphorylation) and numbers, therefore contribute to the increased EPSP amplitude after HFS.

Long-term depression

Lynch et al. **[189]** first observed lasting activity-dependent heterosynaptic depression of synaptic transmission in the hippocampus as a reversible depression in the non-tetanically stimulated pathway. There are two forms of LTD, homosynaptic and heterosynaptic depression. Homosynaptic depression is induced only in the pathway that receives the induction protocol, whereas heterosynaptic depression describes a form of depression in a pathway that has not been directly subjected to the induction protocol. Homosynaptic LTD in the hippocampus is of particular relevance to the subject of this article. In contrast to LTP, long-term depression is characterized by a lasting decrease in synaptic efficacy following specific forms of electrical stimulation. It is usually induced *in vitro* by low frequency stimulation (600-900 pulses at 1 Hz) **[190, 191]**. However, LTD induction with this protocol becomes more difficult with increasing age of the donor animal, and LTD cannot be induced in adult (~after P40) rats **[192, 193].** Instead, 1 Hz trains of 900 paired pulses (50 ms interpulse interval) or trains of stimuli at a frequency of 5-10 Hz are required to induce LTD in the adult animal **[194, 195]**. The reason for the switch in LTD induction with increasing age is not known at present.

Induction of LTD depends on activation of either NMDA or metabotropic glutamate receptors **[192, 194, 196]** or, in some cases, on the activation of both **[196]**. In LTD, EPSPs represent AMPA receptor-mediated responses; the reduction in EPSPs seems to be the consequence of a decrease in the number of AMPA receptors at the synaptic surface and/or their reduced function as a result of their phosphorylation/de-phosphorylation.

Determinants of LTP vs. LTD

Increases in intracellular postsynaptic Ca^{2+} concentrations are implicated in both LTP and LTD **[34, 197].** It is thought that the particular properties of the Ca^{2+} signal (temporal, spatial or magnitude) play a decisive role in whether LTP or LTD is induced **[198]**. In general, the greater the rise in intracellular Ca^{2+} levels, the more likely it is that LTP will be induced; the likelihood of LTD is higher when smaller intracellular Ca^{2+} rises occur upon stimulation. Activation and de-activation of the CaMKII seems to be a crucial mechanism in LTP and LTD induction, although other kinases, such as protein kinase A and protein kinase C, are also implicated in inducing synaptic plasticity **[199, 200, 201, 202]**. In certain conditions, synaptic plasticity can be induced in a Ca^{2+}-independent fashion **[203]**. Relevant to this review is the fact that stress seems to facilitate LTD induction in adult rats when the protocol used for inducing LTD in young animals is applied **[204]**; this phenomenon has been attributed to the activation of nGR and resultant increases in protein synthesis **[205]**. LTP induction, on the other hand, is impaired after stress, an effect that has also been attributed to nGR activation **[204, 206]**.

NMDA and AMPA receptors and stress

Most excitatory synapses in the brain use the neurotransmitter glutamate to process information transferred between neurons. Fast transmission usually activates a mixture of NMDA and AMPA receptors in the postsynaptic cell; NMDA and AMPA receptors are ionotropic glutamatergic receptors. The unique properties that distinguish NMDA receptors from other ligand-gated ionotropic receptors include the fact that they display a voltage-dependent Mg^{2+} block, have high permeability to Ca^{2+} ions, require extracellular co-activators such as glycine or D-serine, and have slow rise and decay times.

NMDA receptors are ‘silent’ at resting membrane potential, but are crucial for the induction of LTP and LTD. NMDA receptors are built from subunit tetramers. Each receptor comprises two NR1 subunits and two NR2 subunits, each of the latter having very different kinetic properties with regard to decay time, conductance and magnesium

sensitivity **[207, 208, 209, 210]** which define the overall properties of the particular NMDA receptor. The NR2A and NR2B subunits are mainly expressed in forebrain structures such as the cerebral cortex, the hippocampus and the striatum; expression of the NR2C subunit expression is mainly confined to the cerebellum **[146, 211]**, and the NR2D subunit is mainly expressed in the diencepahlon, mesencephalon and brainstem **[212]**. Little is known about the expression and function of the NR3A subunit. Most studies on synaptic plasticity focus on the differential effects NR2A and NR2B as these subunits are predominantly expressed at postsynaptic sites in the forebrain. Importantly, expression of the NR2 subunit is developmentally regulated: NR2B is expressed at higher levels than NR2A during the early postnatal period; this ratio gradually reverses with age. As compared to NR2A-containing receptors, those showing a predominance of NR2B display slower kinetics (significantly longer decay currents and higher Ca^{2+} conductance). Since Ca^{2+} is an important intracellular messenger, different NMDAR-dependent signaling cascades become activated, depending on the dynamics of intracellular Ca^{2+} fluxes. In addition, the NR2A and NR2B subunits apparently bind modulatory kinases in a differential manner, leading to activation of distinct signaling pathways and different modes of synaptic transmission. For example, it has been proposed that CaMKII binds the NR2B subunit with a higher affinity than the NR2A subunit **[213]**. Evidence suggests that predominant activation of the NR2B subunit promotes LTD whereas LTP results from predominant activation of the NR2A subunit. Although the synaptic ratio of NR2A to NR2B subunits is tightly regulated, it can be subject to modulation by neuronal activity **[214]**.

In the overall context of this review, it is worth noting that, Ca^{2+} influx through the different NR2 subunits occurs in response to acute stress, suggesting that stress induces synaptic plasticity. Indeed, studies have shown that the effects of stress are preferentially mediated by NR2B-containing NMDA receptors (see main text).

AMPA glutamate receptors (AMPARs) mediate most excitatory (depolarizing) currents in conditions of basal neuronal activity, and hence, have a major influence on the strength of the synaptic response. These receptors are components of excitatory synapses. Their addition and removal from the surface of the synapse is tightly regulated; insertion of

AMPA receptors at the surface is associated with LTP, whereas their internalization represents LTD. AMPA receptors are hetero-oligomeric complexes (possibly tetramers) and are composed of different combinations of GluR1, GluR2, GluR3, and GluR4 subunits **[215]**. In the mature hippocampus, most AMPA receptors occur as dimers of GluR1 and GluR2 or GluR2 and GluR3; GluR4-containing AMPA receptors are expressed mainly in early postnatal development. The inclusion of the GluR2 subunit renders AMPA impermeable to Ca^{2+} **[216, 217]**. Notably, the majority of AMPA receptors in the hippocampus are GluR2-containing oligomers, i.e. Ca^{2+}-impermeable, an important factor insofar as Ca^{2+} is a crucial and tightly regulated intracellular messenger, whose concentrations determine synaptic efficacy through the modulation of various signaling cascades. The trafficking of AMPA receptors from their sites of synthesis in the cytoplasm into synapses is a regulated process that depends on NMDAR activation. AMPA receptor oligomers are formed in the endoplasmic reticulum in a manner that seems to depend on interactions between the N-terminal domains of the individual subunits **[218]**. GluR1-GluR2 hetero-oligomers are rapidly trafficked from the endoplasmic reticulum and are gylcosylated in the Golgi apparatus. In contrast, GluR2-GluR3 oligomers exit the endoplasmic reticulum more slowly **[219]**. The transport of AMPA receptors into dendrites seems to depend on the microtubular cytoskeleton, powered by motor proteins of the kinesin and dynein superfamilies, with Glutamate Receptor Interacting Protein 1/AMPAR Binding Protein (GRIP1/ABP) serving to link the heavy chain of kinesin with the C-terminal PDZ motif of GluR2 and GluR3 **[220, 221]**.
